# Prognostic prediction and comparison of three staging programs for patients with advanced (T2-T4) esophageal squamous carcinoma after radical resection

**DOI:** 10.3389/fonc.2024.1376527

**Published:** 2024-06-27

**Authors:** Zhongshuai Wang, Feng Li, Mingchuang Zhu, Tao Lu, Linqi Wen, Shengzhao Yang, Xiaofei Zhuang, Shuangping Zhang, Yong Ma, Jianhong Lian

**Affiliations:** ^1^ Cancer Hospital Affiliated to Shanxi Medical University/ Shanxi Province Cancer Hospital/ Shanxi Hospital Affiliated to Cancer Hospital, Chinese Academy of Medical Sciences, Taiyuan, China; ^2^ Shanxi Province Cancer Hospital/ Shanxi Hospital Affiliated to Cancer Hospital, Chinese Academy of Medical Sciences/Cancer Hospital Affiliated to Shanxi Medical University, Taiyuan, China

**Keywords:** esophageal squamous cell carcinoma, prognosis, log odds of positive lymph nodes, nomogram, overall survival

## Abstract

**Purpose:**

Lymph node-based staging protocols are frequently employed to evaluate the prognosis of esophageal cancer, yet their accuracy remains contentious. The present study was conducted to assess the prognostic significance of three lymph node staging systems, namely N stage, lymph node rate (LNR), and log odds of positive lymph nodes (LODDS), in patients diagnosed with advanced (T2-T4) esophageal squamous cell carcinoma (ESCC).

**Methods:**

This cohort comprised 319 eligible patients, with an additional 409 individuals retrieved from the Surveillance, Epidemiology, and End Results (SEER) database, forming the validation cohort. Differences in overall survival (OS) of patients between groups were assessed using the log-rank test. Prognostic independent risk variables were identified, and lymph nodes (LN) prognostic models were built using multivariate Cox regression analysis. Besides, the predictive accuracy of each model was evaluated utilizing the (-2) log-likelihood ratio (-2LLR), the likelihood ratio χ2 score (LRχ2), the Akaike information criterion (AIC), and Harrell’s concordance index (C-index). To further evaluate the potential superiority of the model, a nomogram was constructed for comparison with the conventional Tumor Node Metastasis (TNM) staging approach.

**Results:**

Independent prognostic factors for advanced ESCC include the N stage, LNR, and LODDS. Herein, LODDS presented higher values for C-index and LRχ2, and lower values for AIC and -2LLR in OS compared to the others. Consequently, a nomogram was constructed based on LODDS. Calibration curves exhibited strong agreement, and assessment through C-index, receiver operating characteristic (ROC) curves, and clinical decision curve analysis (DCA) demonstrated promising clinical applicability.

**Conclusion:**

LODDS emerges as a promising future prognostic indicator. After surgery, the proposed model holds the potential to provide valuable treatment recommendations for patients with advanced ESCC.

## Introduction

One of the most common gastrointestinal malignant tumors, esophageal cancer (EC), features a high global rate of morbidity and death. Globally, there were approximately 604,000 new cases of esophageal cancer in 2020, accounting for 3.1% of all malignant tumors and ranking the eighth. Approximately 544,000 related deaths occurred, occupying 5.5% of all malignant tumors and ranking the sixth (1). Adenocarcinoma and esophageal squamous carcinoma are the two main types of esophageal cancer, with the latter more prevalent in Western nations and the former more common in Asian regions (2, 3). China faces a significant public health issue with a high incidence of esophageal cancer, which has mortality and incidence rates greater than the global average (4, 5). In the modern world, the primary treatment for resectable esophageal cancer is radical resection of the malignancy in conjunction with dissection of lymph nodes. Nevertheless, the survival rate for individuals who have received surgical treatment for esophageal carcinoma still remains relatively modest (6, 7). This is because a significant number of patients already have lymph node metastasis at the time of diagnosis (8). Accurate cancer staging is vital for effective clinical prognostic advice, given the significant impact of lymph node metastasis on the prognosis of postoperative esophageal cancer patients.

The current clinical staging system for esophageal cancer, known as the 8th edition of the Tumor Node Metastasis (TNM) staging system, was introduced by the American Joint Committee on Cancer (AJCC). It relies on the pathological lymphatic number (pN) of individuals diagnosed with esophageal cancer and is extensively utilized in medical practice (9). However, the correlation between the extent of lymph node involvement and the number of lymph node dissections, coupled with the absence of a definitive guideline for esophageal cancer surgery, makes this staging method prone to bias (10). To enhance the precision of forecasting the outcome of postoperative survival in patients with EC, staging systems relying on the quantity or proportion of lymph node dissection should be necessarily taken into account (11).

To enhance the accuracy of predicting patients’ survival prognosis and minimize staging bias, a novel system for staging lymph nodes, known as the positive lymph node count (PLN) and lymph node ratio (LNR), has been suggested for patients with malignant tumors (11, 12). Nevertheless, the association between LNR and postoperative survival remains controversial when individuals with cancerous growths lack lymph node participation or when all excised lymph nodes yield positive LN results (13). In this case, the logarithm of the ratio between PLN and the count of cleared negative lymph nodes, the LODDS has been thoroughly discussed (10, 14).

The LODDS, a newly developed staging system based on rates, performs well in accurately forecasting the outlook of various malignant tumors including non-small cell lung cancer, rectal cancer, breast cancer, gastric cancer, pancreatic cancer, and numerous others (14–16). While there are limited reports on the prognosis of individuals with esophageal cancer after surgery, the LODDS has shown promise in this context as well (10). However, studies on esophageal cancer in China have mainly focused on open surgery, while thoracoscopic minimally invasive surgery has gradually emerged in the past decade. Therefore, effects of the two surgical methods on the long-term survival of patients were hereby considered. In addition, although the depth of tumor invasion is closely related to lymph node metastasis, the probability of lymph node metastasis remains low in T1 stage patients with shallow tumor invasion. Besides, the probability of lymph node metastasis is significantly increased when the degree of tumor invasion goes deep into the submucosal layer (17–19). Moreover, few clinicians perform lymph node dissection for endoscopic submucosal dissection (ESD), the primary surgical method for T1 superficial esophageal cancer. Currently, there are scarce solid data to elucidate the impact of LODDS on the prognosis of patients with advanced ESCC who have undergone R0 resection, or a comprehensive prognostic evaluation scheme incorporating multiple clinical factors.

The main goal of this research was to identify a superior classification system by examining the efficacy of three lymph node staging methods, N, LNR, and LODDS, in assessing the long-term survival prognosis of patients with advanced ESCC who had undergone R0 resection. A single cohort from China was utilized for analysis. Meanwhile, the findings were validated utilizing the Surveillance, Epidemiology, and End Results (SEER) dataset. The aim was to integrate these three staging systems to clarify the favorable outcomes of LODDS in forecasting the extended-term prognosis of individuals diagnosed with advanced ESCC. Furthermore, a nomogram was constructed using the most accurate predictive algorithm to help medical professionals effectively identify patients at higher risk.

## Materials and methods

### Patients criteria

Clinicopathological data from patients with esophageal cancer at Shanxi Hospital of Cancer Hospital, Chinese Academy of Medical Sciences, were collected retrospectively, and 319 were ultimately considered eligible for inclusion in the study. To ensure adequate follow-up time, the study interval ranged from 2015 to 2018. The inclusion criteria met all of the following conditions: 1. patients with complete clinicopathological data; 2. those clinically diagnosed with advanced (T2-T4) esophageal squamous carcinoma; 3. those undergoing radical resection (R0) with the number of intraoperative lymph node dissections ≥ 1; 4. those having received no other tumor-related treatments prior to surgery; 5. those with no distant metastases; and 6. those with survival time of at least greater than three months after surgery. To ensure study feasibility, individuals with esophageal cancer diagnosed with other malignancies were excluded from the study group. All procedures were carried out by experienced thoracic surgeons. Data collected included gender, age, smoking history, drinking history, body mass index (BMI), primary site of tumor, tumor diameter, postoperative radiotherapy and chemotherapy, TNM stage, degree of differentiation, total number of lymph nodes dissected, number of lymph nodes that tested positive, and follow-up information.

**Figure 1:**
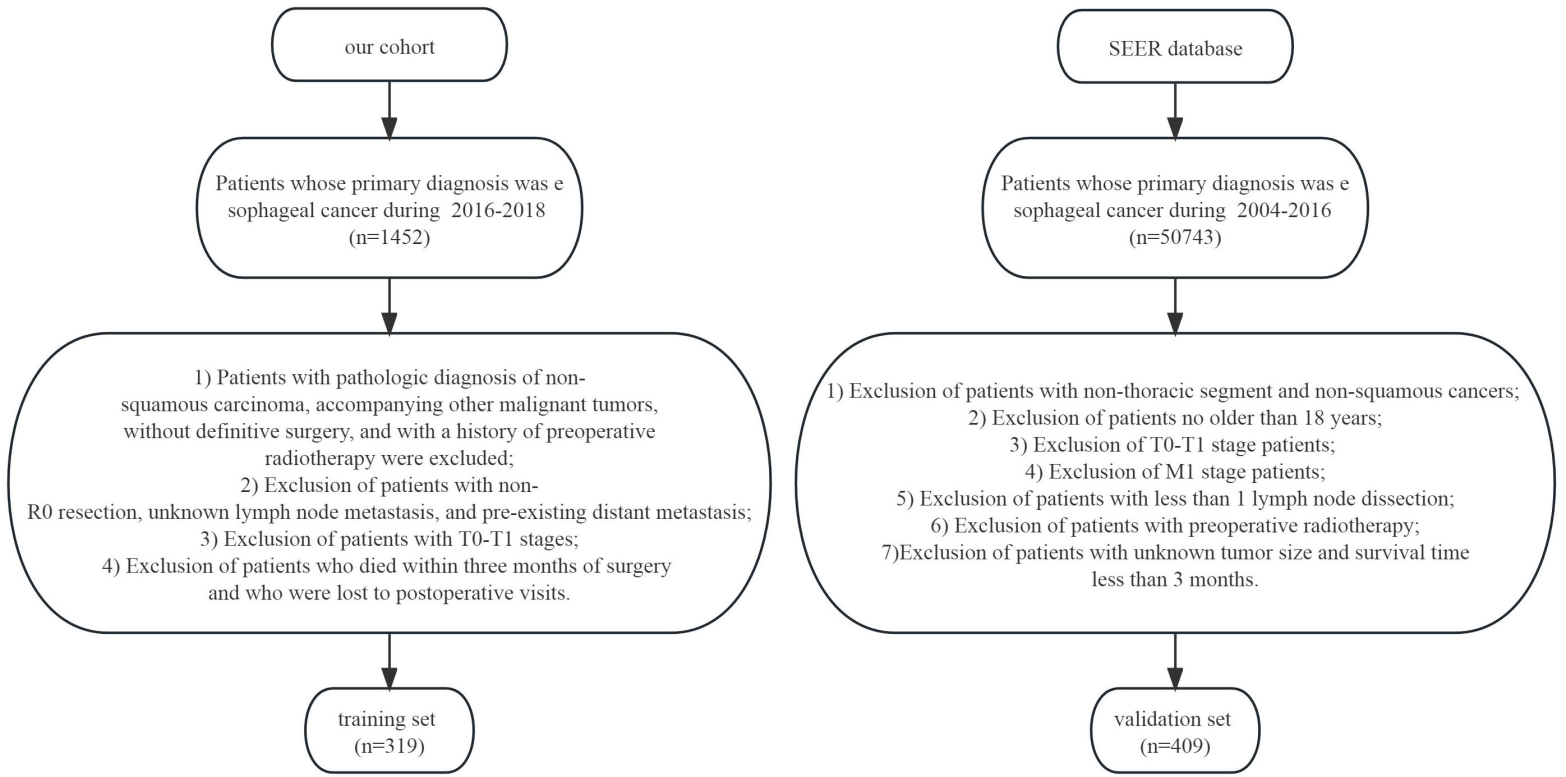
Flowchart for screening.

For validation of the study findings, an additional independent cohort was obtained in the SEER database (https://www.cancer.gov/), with 409 patients with progression (T2-T4) involved. The screening criteria included: 1. Patients with primary tumor diagnosed between 2004 and 2016 in the esophagus; 2. those aged over 18 years; 3. those with advanced (T2-T4) esophageal squamous carcinoma; 4. those who were surgically treated and did not receive preoperative radiotherapy; 5. those with intraoperative lymph node dissection of ≥1 lymph node; 6. those exposed to absence of distant metastasis; and 7. those who survived less than three months. The TNM staging of all patients was updated according to the 9th edition of AJCC criteria. The survival outcome of this research was over survival (OS). **Figure 1** illustrates the patient screening process.

### Determination of various staging protocols and cut-off values based on lymph nodes

The calculation of LODDS involved the utilization of the subsequent equation: log10(PLN+0.5)/(NLN+0.5). Here, PLN represents the count of regionally positive lymph nodes, while NLN denotes the amount of negative lymph nodes. The latter was determined by subtracting the count of positive lymph nodes (PLN) from the total count of dissected lymph nodes (DLN). To avoid irrational numbers, both the numerator and denominator were increased by 0.5. Using the formula PLN/DLN, LNR was defined as the ratio of the count of positive lymph nodes to the total count of cleared lymph nodes. Additionally, the optimal cut-off values for the continuous variables (LNR, LODDS) were determined and processed for categorical grouping using the X-tile software. Within the study group, N staging was determined based on the 8th edition of the AJCC/TNM criteria: N0(0), N1(1–2), N2(3–6), and N3(≥7); LNR was categorized as: LNR0(0), LNR1(≤0.05), LNR2(≤0.24), and LNR3(≤1.00); and LODDS was defined as: LODDS1(≤-1.01), LODDS2(≤-0.49), and LODDS3(≤1.01).

### Statistical analysis

Medians [interquartile range (IQR)] were used to express all continuous variables, while percentages or counts were employed to express categorical variables and hierarchical information. Besides, the Kaplan-Meier (KM) survival analysis was carried out to compute five-year survival rates, and the log-rank test was employed to assess variations among variable groups. Furthermore, the significant study indicators from the univariate analysis were included in the multivariate Cox regression analysis to identify potential risk factors that could independently predict prognosis. Subsequently, maintaining consistency with other confounders, the three study variables N, LNR, and LODDS were independently incorporated into the multivariate Cox regression model to construct Model I (N), Model II (LNR), and Model III (LODDS). Finally, N, LNR, and LODDS were combined to build another model, hereby termed as Model IV. Meanwhile, hazard ratios (HR values) and 95% confidence intervals (CIs) were computed for every variable. To evaluate the fit goodness of each prognostic model, the (-2) log-likelihood ratio (-2LLR), the likelihood ratio χ2 score (LRχ2 test), and the Akaike information criterion (AIC) were calculated. Meanwhile, Harrell’s concordance index (C-index) was employed to assess the prediction accuracy of the model. The model fit, accuracy, and prediction were found to improve with decreasing -2LLR and AIC values and increasing LRχ2 test and C-index values, respectively. Following that, the nomogram was constructed using the model with the best predictive effect, and the degree of fit between the actual and predicted survival of that nomogram was characterized by calibration curves. Concurrently, the clinical significance of the nomogram was evaluated by constructing ROC and DCA. The degree of association between various lymph node staging methods and the results of Spearman correlation analysis (r_s_) was visualized using scatter plots.

Additionally, statistical significance was determined by considering P<0.05 in this research, with IBM SPSS Statistics 21.0 software, X-tile software (version 3.6.1), R software (version 4.2.1), and GraphPad Prism 8 software adopted for statistical analysis.

## Results

### Basic data and clinicopathological features of the patients

**Figure 2:**
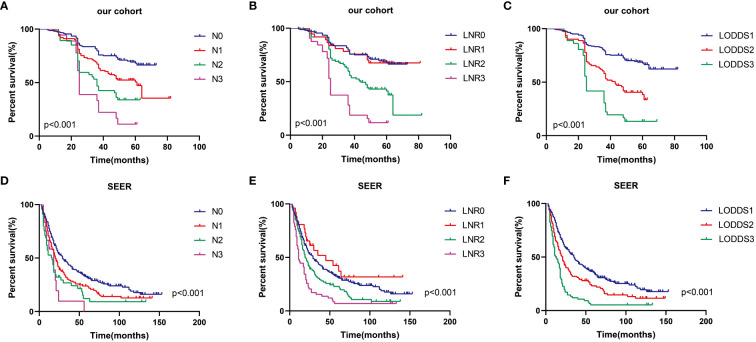
Prognostic impact of three LN systems on OS in advanced ESCC patients in the training set **(A–C)** and validation set **(D–F)**.

**Table 1** presents information regarding the clinical features of the 319 patients with advanced ESCC involved in the present study (as a training set) and the 409 patients with advanced ESCC screened from the SEER database (as a validation set). In the training and validation groups, there were 146 and 312 patients having experienced terminating events, demonstrating 5-year OS survival rates of 54.2% and 23.7%, respectively. In the two cohorts, the median age of patients was 62 (IQR, 56 to 67 years) and 65 (IQR, 58 to 74 years) respectively, with a predominant male representation (62.4% and 59.4%) and females accounting for 37.6% and 40.6% respectively. In terms of primary tumor site, the majority of ESCC patients preferred the middle and lower esophagus. The majority of patients in both cohorts were in the N0 stage, consisting of 153 and 224 patients, which accounted for 48.0% and 54.8% of the total patient count, respectively. Conversely, the N3 stage had the smallest proportion of patients, with only 18 and 12 patients, comprising 5.6% and 2.9% of the overall patient count, respectively. Furthermore, in the training and validation sets, the median DLN values were 18 (IQR, 13 to 27) and 13 (IQR, 6 to 20), the median PLN values were 1 (IQR, 0 to 2) and 0 (IQR, 0 to 1), the median LNR values were 0.030 (IQR, 0.000 to 0.120) and 0.000 (IQR, 0.000 to 0.140), and the median LODDS values were -1.18 (IQR, -1.54 to -0.76) and -1.07 (IQR, -1.46 to -0.66), respectively. Furthermore, the median survival times for the single-center study cohort versus the SEER dataset were 49 months (IQR, 27 to 60 months) and 21 months (IQR, 11 to 51 months), respectively. As shown in **Figure 2**, patients with earlier N, LNR, and LODDS staging demonstrated a considerably higher overall survival rate (total log-rank P<0.001). However, compared with the other two stages, the K-M curves of LODDS are more separated, more balanced, and easier to distinguish. Furthermore, the prognosis was worsened, and patient mortality increased with the increase of the LNR and LODDS levels (**Supplementary Figure 1**).

**Table 1:** Basic clinicopathological information of advanced ESCC patients in the training set and validation set.

Characteristics	Training set (our cohort)			Validation set (SEER)		
	No. of patients n (%)	5-Years OS (%)	P-value	No. of patients n (%)	5-Years OS (%)	P-value
Gender			0.017			0.036
Male	199 (62.4)	48.7		243 (59.4)	19.8	
Female	120 (37.6)	63.3		166 (40.6)	29.5	
Age (years)			0.037			0.045
≤57	98 (30.7)	45.9		99 (24.2)	27.3	
>57	221 (69.3)	57.9		310 (75.8)	22.6	
Race						0.005
White	NR			276 (67.5)	25.0	
Black	NR			74 (18.1)	6.8	
Others	NR			59 (14.4)	39.0	
Smoking history			0.003			
No	155 (48.6)	62.6		NR		
Yes	164 (51.4)	46.3		NR		
Drinking history			0.014			
No	201 (63.0)	59.7		NR		
Yes	118 (37.0)	44.9		NR		
BMI (kg/m^2^)			0.004			
≤25	249 (78.1)	58.2		NR		
>25	70 (21.9)	40.0		NR		
Tumor location			0.780			0.020
Upper	18 (5.6)	55.6		52 (12.7)	17.3	
Middle	188 (58.9)	56.4		154 (37.7)	20.1	
Lower	113 (35.4)	50.4		203 (49.6)	28.1	
Tumor diameter (cm)			0.010			0.090
≤2.5	65 (20.4)	69.2		102 (24.9)	27.5	
>2.5	254 (79.6)	50.4		307 (75.1)	22.5	
Radiotherapy			0.501			0.516
No	245 (76.8)	55.5		310 (75.8)	25.2	
Yes	74 (23.2)	50.0		99 (24.2)	19.2	
Chemotherapy			0.349			0.375
No	232 (72.7)	56.0		278 (68.0)	22.3	
Yes	87 (27.3)	49.4		131 (32.0)	26.7	
Surgical method			0.095			
Open chest surgery	66 (20.7)	45.5		NR		
Minimally invasive surgery	253 (79.3)	56.5		NR		
Differentiation			0.004			0.129
Well	24 (7.5)	79.2		26 (6.4)	34.6	
Medium	80 (25.1)	56.3		203 (49.6)	25.6	
Poorly	96 (30.1)	41.7		168 (41.1)	19.0	
Unknown	119 (37.3)	58.0		12 (2.9)	33.3	
T stage			<0.001			0.003
T2	117 (36.7)	69.2		110 (26.9)	38.2	
T3	193 (60.5)	47.2		270 (66.0)	18.9	
T4	9 (2.8)	11.1		29 (7.1)	13.8	
N stage			<0.001			0.001
N0	153 (48.0)	68.6		226 (55.3)	28.8	
N1	101 (31.7)	49.5		126 (30.8)	19.0	
N2	47 (14.7)	34.0		45 (11.0)	15.6	
N3	18 (5.6)	11.1		12 (2.9)	8.3	
LNR			<0.001			<0.001
LNR0	153 (48.0)	68.6		226 (55.3)	28.8	
LNR1	37 (11.6)	67.6		26 (6.4)	38.5	
LNR2	97 (30.4)	40.2		95 (23.2)	16.8	
LNR3	32 (10.0)	12.5		62 (15.2)	9.7	
LODDS			<0.001			<0.001
LODDS1	202 (63.3)	67.8		223 (54.5)	32.7	
LODDS2	81 (25.4)	38.3		107 (26.2)	16.8	
LODDS3	36 (11.3)	13.9		79 (19.3)	7.6	

P-value, Log-rank test and Kaplan-Meier method were used for univariate survival analysis, and p<0.05 was considered statistically significant; OS, overall survival; LNR, lymph node rate; LODDS, log odds of positive lymph nodes.

### Construction of predictive models

The results of the univariate analysis are shown in **Table 1**. Gender, age, history of smoking, history of drinking, BMI, tumor diameter, T stage, N stage, degree of differentiation, LNR, and LODDS were presented as potential prognostic risk factors for OS (total P<0.05). The multivariate Cox risk-proportional regression model included statistically significant indicators from the univariate analysis, excluding those related to lymph nodes, serving as the modeling foundation. Subsequently, the analysis included N (Model I), LNR (Model II), and LODDS (Model III), respectively. As indicated in **Table 2**, the three LN categorization study indicators were independent risk factors for esophageal cancer patients’ prognosis. Finally, all four LN model variables mentioned above were incorporated in Model IV to determine differences between them. Additionally, it should be noted that in Model IV, N stage and LNR demonstrated less effectiveness compared to LODDS and were not statistically significant independently.

**Table 2:** Multivariate Cox regression analysis for OS and predictive performance of different LNs models in the training set (N=319).

Variable	Model I (N)		Model II (LNR)		Model III (LODDS)		Model IV	
	HR(95%CI)	P-value	HR(95%CI)	P-value	HR(95%CI)	P-value	HR(95%CI)	P-value
Gender		0.951		0.749		0.887		0.862
Male	Ref		Ref		Ref		Ref	
Female	0.984 (0.582–1.663)		0.917 (0.540–1.557)		0.963 (0.569–1.630)		0.954 (0.559–1.627)	
Age (years)		0.523		0.540		0.511		0.613
≤57	Ref		Ref		Ref		Ref	
>57	0.891 (0.626–1.269)		0.896 (0.631–1.272)		0.889 (0.628–1.261)		0.913 (0.640–1.301)	
Smoking history		0.139		0.118		0.124		0.111
No	Ref		Ref		Ref		Ref	
Yes	1.507 (0.875–2.595)		1.556 (0.894–2.708)		1.528 (0.890–2.622)		1.564 (0.902–2.710)	
Drinking history		0.607		0.830		0.421		0.399
No	Ref		Ref		Ref		Ref	
Yes	1.125 (0.719–1.760)		1.050 (0.672–1.643)		1.200 (0.769–1.874)		1.221 (0.768–1.942)	
BMI (kg/m^2^)		0.001		<0.001		<0.001		<0.001
≤25	Ref		Ref		Ref		Ref	
>25	1.894 (1.303–2.753)		1.961 (1.347–2.857)		1.980 (1.361–2.881)		2.020 (1.384–2.947)	
Tumor diameter (cm)		0.253		0.436		0.375		0.393
≤2.5	Ref		Ref		Ref		Ref	
>2.5	1.337 (0.813–2.197)		1.220 (0.740–2.009)		1.252 (0.762–2.059)		1.244 (0.754–2.052)	
Differentiation		0.113		0.095		0.087		0.068
Well	Ref		Ref		Ref		Ref	
Medium	1.715 (0.664–4.427)		1.851 (0.717–4.778)		2.183 (0.840–5.675)		2.217 (0.835–5.887)	
Poorly	2.263 (0.892–5.738)		2.380 (0.942–6.017)		2.681 (1.055–6.816)		2.831 (1.086–7.381)	
Unknown	1.507 (0.590–3.850)		1.577 (0.619–4.019)		1.826 (0.715–4.663)		1.878 (0.718–4.912)	
T stage		0.005		0.012		0.042		0.027
T2	Ref		Ref		Ref		Ref	
T3	1.526 (1.030–2.259)		1.419 (0.952–2.113)		1.355 (0.907–2.023)		1.343 (0.898–2.008)	
T4	3.833 (1.618–9.080)		3.448 (1.497–7.942)		2.812 (1.233–6.415)		3.178 (1.351–7.476)	
N stage		<0.001						0.552
N0	Ref						Ref	
N1	1.742 (1.160–2.616)						1.593 (0.403–6.292)	
N2	2.283 (1.422–3.665)						1.075 (0.273–4.237)	
N3	2.873 (1.532–5.388)						1.080 (0.274–4.263)	
LNR				<0.001				0.617
LNR0			Ref				Ref	
LNR1			0.975 (0.514–1.852)				0.658 (0.149–2.905)	
LNR2			2.141 (1.435–3.196)				0.978 (0.268–3.576)	
LNR3			3.672 (2.189–6.160)					
LODDS						<0.001		0.035
LODDS1					Ref		Ref	
LODDS2					2.350 (1.587–3.479)		1.940 (1.076–3.496)	
LODDS3					3.729 (2.319–5.995)		3.600 (1.012–12.801)	
C-index	0.697		0.712		0.719			
LRχ2	73.93		86.62		90.89			
-2LLR	1524.04		1512.21		1508.24			
AIC	1543.82		1531.13		1524.86			

CI, confidence interval; HR, hazard ratio; C-index, Harrell’s concordance index; LRχ2, the likelihood ratio χ 2 score; -2LLR, the (-2) log-likelihood ratio.

AIC, the Akaike information criterion.

In the SEER validation set, the three LN classification systems indicated similar results to the single-center cohort. Among them, the LODDS staging system was similarly superior to N with LNR in terms of prognostic efficiency for ESCC (**Supplementary Table 1**).

### Comparative prognostic efficiency of LN staging systems

Variations in the predictive ability of the three lymph node stages were evaluated by computing the (-2) log-likelihood ratio (-2LLR), the likelihood ratioχ2 score (LRχ2), the Akaike information criterion (AIC), and Harrell’s concordance index (C-index) values. As shown in **Table 2** and **Supplementary Table 1**, LODDS exhibited the lowest values of -2LLR and AIC (training set: 1508.24, 1524.86 vs. validation set: 3247.71, 3255.87), while its LRχ2 and C-index values (training set: 90.89, 0.719 vs. validation set: 83.76, 0.0.655) were the highest. This demonstrated that compared to the N and LNR staging systems, the prognostic effectiveness of the LODDS was higher for patients with advanced esophageal cancer following R0 resection. Additionally, Model IV indicated no notable distinction between N stage and LNR, implying that the LODDS staging system might offer a more effective approach to classifying LN.

### Correlation between different lymph node classifications

**Figure 3:**
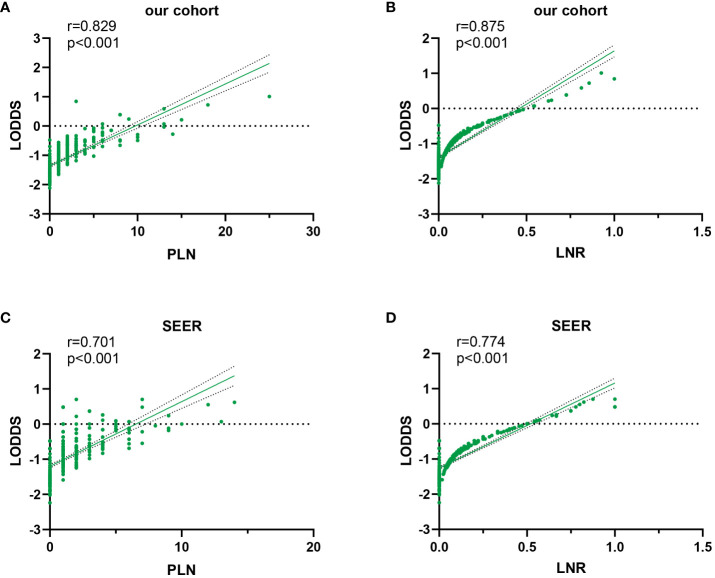
Scatter plot of LODDS versus PLN and LNR in the training set **(A, B)** and validation set **(C, D)**.

In this study, scatter plots were drawn to illustrate the superiority of LODDS over alternative LN staging options by giving a comprehensive and integrated depiction of their correlation. The results of the Spearman correlation analysis indicated that the relationship between LODDS and LNR exhibited greater strength compared to that between LODDS and PLN (training set: r_s_=0.875 vs. r_s_=0.829, validation set: r_s_ =0.774 vs. r_s_=0.701), and this disparity was statistically significant (p<0.001). In **Figure 3**, it displayed the positive correlation between LODDS and the two other staging schemes. Among these, the relationship between LODDS and LNR was the closest to being linear, with LODDS presenting an increasing trend as LNR increased. However, when LNR took the value of 0, the corresponding LODDS values were unevenly distributed, suggesting a greater superiority of LODDS in distinguishing heterogeneity. Furthermore, LODDS showed greater variability at the same PLN value when PLN<10, indicating the constantly heterogeneous survival outcomes of the patients.

### LODDS subgroup analysis

Furthermore, variables in the research cohort were classified into LODDS subgroups based on the results of the univariate analysis. As demonstrated by **Table 3**, there were 202 (63.3%) patients in the LODDS1 group, 81 (25.4%) in the LODDS2 group, and 36 (11.3%) in the LODDS3 group. Patients in the LODDS3 group showed higher LNR values, deeper tumor invasion (T), bigger tumor diameters, and a higher risk of local lymph node metastases (N) compared to the LODDS1 group (all of the above variables P< 0.05). In the SEER dataset, those in the LODDS3 group demonstrated comparable performance to the training set concerning tumor invasion, the number of local lymph node metastases, and LNR values. The only exception was tumor diameter, where no statistically significant difference was observed (**Supplementary Table 2**).

**Table 3:** Subgroup analysis stratified by the LODDS system in the training set.

Variable	Total	LODDS1	LODDS2	LODDS3	Chi-square	P value
Total	319	202 (63.3)	81 (25.4)	36 (11.3)		
Gender					1.450	0.484
Male	199 (62.4)	130 (64.4)	46 (56.8)	23 (63.9)		
Female	120 (37.6)	72 (35.6)	35 (43.2)	13 (36.1)		
Age (years)					7.869	0.020
≤57	98 (30.7)	54 (26.7)	26 (32.1)	18 (50.0)		
>57	221 (69.3)	148 (73.3)	55 (67.9)	18 (50.0)		
Smoking history					6.299	0.043
No	155 (48.6)	91 (45.0)	49 (60.5)	15 (41.7)		
Yes	164 (51.4)	111 (55.0)	32 (39.5)	21 (58.3)		
Drinking history					5.718	0.057
No	201 (63.0)	120 (59.4)	60 (74.1)	21 (58.3)		
Yes	118 (37.0)	82 (40.6)	21 (25.9)	15 (41.7)		
BMI (kg/m^2^)					0.515	0.773
≤25	249 (78.1)	160 (79.2)	61 (75.3)	28 (77.8)		
>25	70 (21.9)	42 (20.8)	20 (24.7)	8 (22.2)		
Tumor diameter (cm)					8.193	0.017
≤2.5	65 (20.4)	50 (24.8)	13 (16.0)	2 (5.6)		
>2.5	254 (79.6)	152 (75.2)	68 (84.0)	34 (94.4)		
Differentiation					-0.024*	0.764
Well	24 (7.5)	17 (8.4)	4 (4.9)	3 (8.3)		
Medium	80 (25.1)	52 (25.7)	22 (27.2)	6 (16.7)		
Poorly	96 (30.1)	51 (25.2)	31 (38.3)	14 (38.9)		
Unknown	119 (37.3)	82 (40.6)	24 (29.6)	13 (36.1)		
T stage					0.591*	<0.001
T2	117 (36.7)	95 (47.0)	20 (24.7)	2 (5.6)		
T3	193 (60.5)	105 (52.0)	58 (71.6)	30 (83.3)		
T4	9 (2.8)	2 (1.0)	3 (3.7)	4 (11.1)		
N stage					0.926*	<0.001
N0	153 (48.0)	145 (71.8)	7 (8.6)	1 (2.8)		
N1	101 (31.7)	54 (26.7)	44 (54.3)	3 (8.3)		
N2	47 (14.7)	3 (1.5)	27 (33.3)	17 (47.2)		
N3	18 (5.6)	0 (0.0)	3 (3.7)	15 (41.7)		
LNR					0.955*	<0.001
LNR0	153 (48.0)	145 (71.8)	7 (8.6)	1 (2.8)		
LNR1	37 (11.6)	37 (18.3)	0 (0.0)	0 (0.0)		
LNR2	97 (30.4)	20 (9.9)	74 (91.4)	3 (8.3)		
LNR3	32 (10.0)	0 (0.0)	0 (0.0)	32 (88.9)		

*Gamma value, two-way ordered chi-square test.

### Nomogram construction and clinical practicability analysis

**Figure 4:**
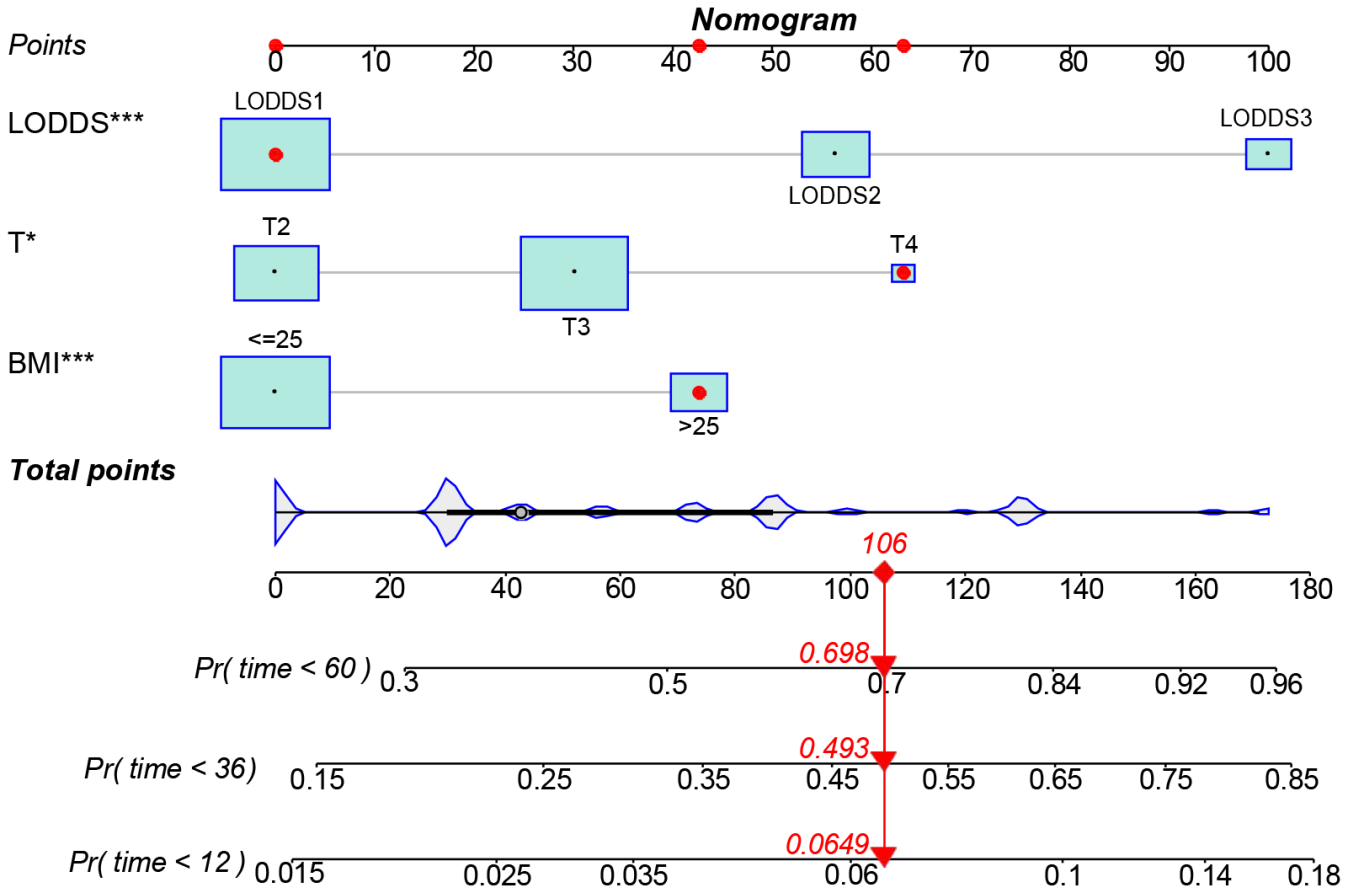
A nomogram for predicting the 1-, 3-, and 5-year OS for advanced ESCC patients. *The independent variables of the model were significantly correlated with the prognosis. (*: p<0.05, **: p<0.01, ***<0.001).

**Figure 5:**
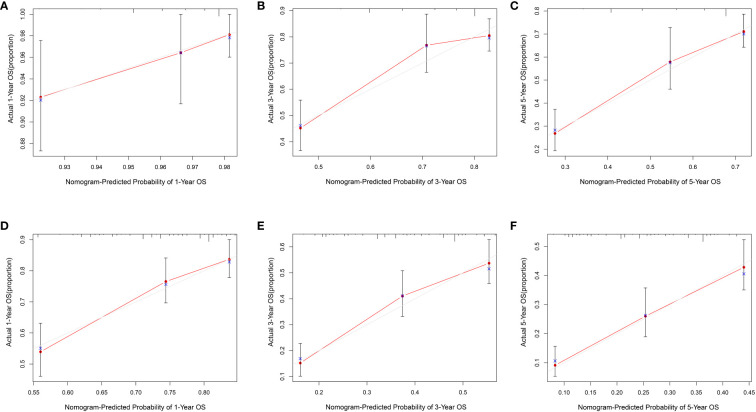
The calibration curves for 1-, 3- and 5- year ESCC patients in the training set **(A–C)** and validation set **(D–F)**.

**Figure 6:**
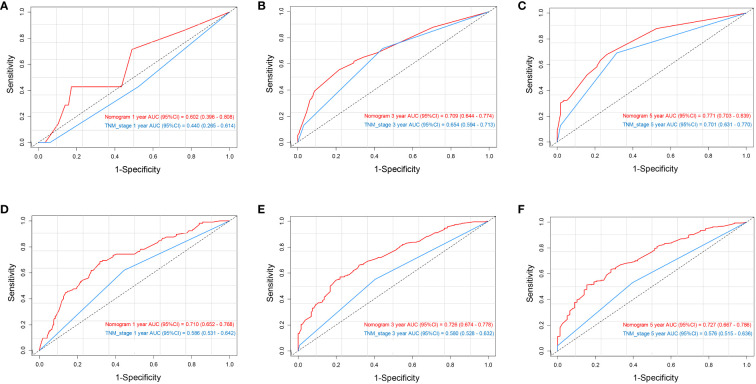
ROC analysis of 1-, 3-, and 5-year OS based on the nomogram and TNM stage in the training set **(A–C)** and validation set **(D–F)**.

**Figure 7:**
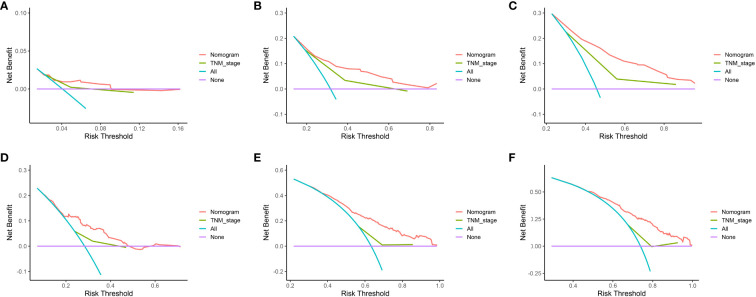
DCA of 1-, 3-, and 5-year OS based on the nomogram and TNM stage in the training set **(A–C)** and validation set **(D–F)**.

The data above indicated the better prediction efficacy of the LODDS staging system than the other two LN staging systems. Based on a multivariate Cox model including LODDS, a nomogram was further created to predict 1-, 3-, and 5-year OS, as depicted in **Figure 4**. The training set contained variables for T stage, LODDS, and BMI, while factors supported by the nomogram for the SEER database validation set included T stage, LODDS, gender, age, race, and tumor site (**Supplementary Figure 2**). The present work was internally evaluated by Bootstrap utilizing the sampling approach, with C-index values of 0.719 and 0.655 in the training and validation sets, respectively. Furthermore, as depicted in **Figure 5**, the calibration plots for the probability of 1-, 3-, and 5-year OS exhibited favorable concordance between the observed survival status and the predicted survival rate by the nomogram in both the training and validation sets. ROC curves were plotted to measure the accuracy of 1-, 3-, and 5-year OS prediction efficacy (**Figure 6**). The area under the curve (AUC) values in the training cohort were 0.602, 0.709, and 0.771, respectively, and 0.710, 0.726, and 0.727 in the validation cohort. Subsequently, the DCA was created to further validate the nomogram’s clinical value (**Figure 7**). ROC and DCA were used to compare the nomogram with the TNM stage. In both the training and validation sets, the ROC results indicated that the nomogram outperformed the TNM stage in predicting OS. Meanwhile, DCA showed that compared to the TNM stage, the LODDS-based model demonstrated superior clinical utility across various risk thresholds, indicating its enhanced performance.

## Discussion

Accurate evaluation of patients’ lymph node status holds considerable significance in managing and post-surgery monitoring of esophageal cancer. The LODDS staging system, known for its effective prognostic prediction in various cancers (15, 20, 21), is a relatively new approach to lymph node staging. However, research on its prognostic effectiveness for advanced esophageal squamous cell carcinoma is limited. Herein, a COX model was developed to identify and validate LODDS as an independent prognostic factor for advanced ESCC patients after R0 surgery, and its performance in predicting long-term survival prognosis was confirmed to be better than that of N, LNR staging systems.

Accurate lymph node staging is paramount in clinical settings as it directly impacts both surgical outcomes and long-term prognosis for individuals with esophageal cancer. Currently, the 8th edition of the TNM staging system published by the AJCC/UICC remains the globally accepted standard for staging esophageal cancer. Despite updates, the lymph node staging criteria, primarily based on the number of lymph node metastases, have seen minimal changes since the 7th edition. However, studies have shown a significant association between the overall quantity of lymph nodes removed during the surgical procedure and the quantity of metastatic lymph nodes detected through postoperative pathology. Insufficient lymph node dissection can indeed result in “staging bias” (22–24). Hence, numerous academics and investigators have sought innovative methods to classify lymph nodes, considering both the quantity of lymph nodes examined and other factors, to assess the long-term prognosis of individuals with esophageal carcinoma post-surgery. These methods include evaluating the number of negative lymph nodes (NLN) (25), the ratio of negative to positive lymph nodes (RNP) (26), the lymph node ratio (LNR) (27), and the log odds of positive lymph nodes (LODDS) (28).

Prior research has indicated the better performance of the LNR staging system in terms of the scope of LN metastasis and the forecast of the long-range prognosis of individuals with esophageal cancer in comparison to the N staging system due to its independence from the overall count of dissected lymph nodes (29). Specifically, Zhang H et al. (27) examined the survival of 387 patients with ESCC, and discovered that the LNR staging and the suggested tumor-ratio-metastasis (TRM) staging exhibited significant advantages in forecasting the OS of ESCC patients compared to the conventional N staging and TNM staging. However, the predictive efficacy of LNR for survival prognosis remains contentious when patients show either no lymph node involvement or complete lymph node involvement (30). Furthermore, several studies have found that the LODDS staging system may be more effective at prognostic prediction than the LNR staging system (28, 31). Many people hold that the LODDS value can be utilized as a new prognostic index, and that it is more accurate than the existing relevant standards for various tumors (15, 21). However, its accuracy in esophageal cancer remains controversial. LODDS holds promising advantages in predicting patient outcomes by considering both the number of DLN and the influence of NLN. To prevent irrational numbers, 0.5 can be added to both the numerator and denominator.

Liu DT et al. (10) examined 1,667 pT3 stage ESCC patients having undergone esophagectomy, and confirmed that LODDS was more reliable and accurate than N staging and LNR staging in predicting the prognosis of ESCC patients with regard to 5-year overall survival. However, Liu’s study found a lack of option in surgical access and postoperative adjuvant treatment profile, and it was made solely for patients with stage T3 esophageal squamous carcinoma, without taking into account the general status of patients with advanced stages (T2-T4). On this basis, the present research to carried out to expand the evolution of esophageal cancer, examine the components of surgical protocols as well as postoperative radiotherapy and chemotherapy, then study the predictive value of LODDS and other schemes, and conduct further verification in a large cohort. This pioneering study of esophageal cancer in the Taihang Mountains region of China, a high-risk area for the disease, demonstrated LODDS staging as a sensitive approach to staging. Concurrently, the SEER database was employed to validate the continued relevance of the LODDS classification in prognosticating patients with esophageal cancer, and the findings were proven reliable.

To investigate the predictive significance of the N, LNR, and LODDS staging systems, a study group consisting of 319 patients diagnosed with advanced ESCC were involved. The analysis of a single variable indicated a strong correlation between the three lymph node staging systems and the 5-year overall survival rate of patients with EC. The lower the 5-year OS value of EC patients, the greater the subgroup value. According to multivariate analysis, all three LN staging methods were independent prognostic risk factors for ESCC. However, when the three staging systems (N, LNR, and LODDS) were combined into a single model, only LODDS remained significant. This result was replicated in the SEER validation cohort, confirming that LODDS outperformed N and LNR stages in predicting the prognosis of ESCC patients. Furthermore, the superiority of LODDS staging for prognostic efficacy was observed to be better reflected when a small number of DLNs were used. For example, Patient A, with only 1 lymph node dissected (PLN=1), exhibited a superior prognosis (LODDS_A_=0.477), compared to Patient B who had 10 lymph nodes dissected (PLN=10) and a higher LODDS value (LODDS_B_=1.322), despite both having an equivalent LNR of 1. When an insufficient number of lymph nodes were removed, the accuracy of LNR was compromised due to the correlation between the total number of DLN and the number of PLN. Especially in patients without lymph node metastases (PLN=0, LNR=0), neither N staging nor LNR staging might accurately reflect the difference in patient survival. As a result, some experts have advocated that LODDS be considered the best prognostic predictor without taking into account the amount of DLN (28).

When evaluating the model, two aspects were typically considered. On the one hand, indicators such as AIC, -2LLR, and LRχ2test were utilized to measure the model’s goodness-of-fit, while on the other, the C-index represented the accuracy of the model’s predictions. Among them, the smaller the values of AIC and -2LLR, and the higher the values of LRχ2test and C-index, the greater the model’s predictive efficacy. The model’s prognostic effectiveness, however, was poor. In this study, the AIC and -2LLR values of the LODDS model were 1524.86 and 1508.24, respectively, both lower than those of the N and LNR models (1543.82 and 1524.04, 1531.13 and 1512.21). Furthermore, the C-index value of the LODDS staging model exceeded those of the N and LNR staging models (0.719 vs. 0.697 and 0.712, respectively). Hence, it was hereby validated that LODDS surpassed the N and LNR staging methods in forecasting the extended-term endurance of individuals with progressive ESCC.

To build the nomogram, the model with the best predictive performance (LODDS) was utilized. In the training set, the nomogram achieved a C-index of 0.719, demonstrating good accuracy. Similarly, in the validation set, the C-index was 0.655, further confirming its accuracy in both sets. Furthermore, the patients’ 1-, 3-, and 5-year calibration curves after surgery essentially coincided with the 45° dashed line in the training and validation sets, indicating the nomogram’s good accuracy and dependability. Meanwhile, ROC was made to determine the accurate predictive power of 1-, 3-, and 5-year OS. The findings showed that in predicting the postoperative OS of ESCC patients, the nomogram outperformed the conventional TNM stage. Meanwhile, DCA showed comparable outcomes. Additionally, the nomogram demonstrated greater net benefits under the majority of risk thresholds, indicating its powerful clinical applicability.

The current research findings are clinically meaningful. However, there are still several limitations. To begin with, the present study was retrospective and based on a relatively small sample size, which limited the scale of certain research components during statistical analysis. This diminutive scale weakened the overall power of the study. Additionally, the validation cohort was derived from the SEER database, revealing differences in clinical and pathological characteristics compared to the cohort, thus resulting in distinctions in the variables incorporated throughout the various research institutions. Simultaneously, the nomogram developed should be further validated in real-world multi-center and large-sample cohorts. Most significantly, the primary concern still revolves around the debate regarding the applicability of our study findings to clinical patients, as there are currently no established classification standards for LODDS.

## Conclusion

In conclusion, the present research validated the efficacy of the N, LNR, and LODDS classification methods in assessing the survival outlook for individuals diagnosed with advanced (T2-T4) ESCC. Nevertheless, LODDS surpassed the other two LN staging methods, especially in individuals having undergone inadequate lymph node dissection. LODDS was confirmed to have the potential to be a promising prognostic biomarker for patients with advanced esophageal cancer. Besides, a nomogram was created and verified to forecast the overall survival of patients with advanced ESCC based on the new indicator, LODDS. Overall, LODDS shows considerable promise as a valuable tool for clinicians, aiding in postoperative prognosis assessment and facilitating tailored treatment strategies to enhance patient outcomes.

## Data availability statement

The original contributions presented in the study are included in the article/**Supplementary Material**. Further inquiries can be directed to the corresponding authors.

## Ethics statement

The studies involving humans were approved by Ethics Committee of Shanxi Cancer Hospital, Chinese Academy of Medical Sciences (Shanxi Cancer Hospital). The studies were conducted in accordance with the local legislation and institutional requirements. Written informed consent for participation was not required from the participants or the participants’ legal guardians/next of kin in accordance with the national legislation and institutional requirements.

## Author contributions

ZW: Conceptualization, Methodology, Validation, Writing – original draft, Writing – review & editing. FL: Validation, Formal analysis, Writing – review & editing. MZ: Conceptualization, Methodology, Writing – review & editing. TL: Formal analysis, Writing – review & editing. LW: Formal analysis, Writing – review & editing. SY: Supervision, Writing – review & editing. SZ: Supervision, Writing – review & editing. XZ: Supervision, Writing – review & editing. YM: Writing – review & editing. JL: Funding acquisition, Supervision, Writing – review & editing.
