# Supplementary material for: Prognostic prediction and comparison of three staging programs for patients with advanced (T2-T4) esophageal squamous carcinoma after radical resection
Source: Front Oncol. 2024 Jun 27;14:1376527. doi: 10.3389/fonc.2024.1376527 (PMC11236680; doi:10.3389/fonc.2024.1376527)
Supplement: Supplementary file 1 [file DataSheet_1.doc]

**
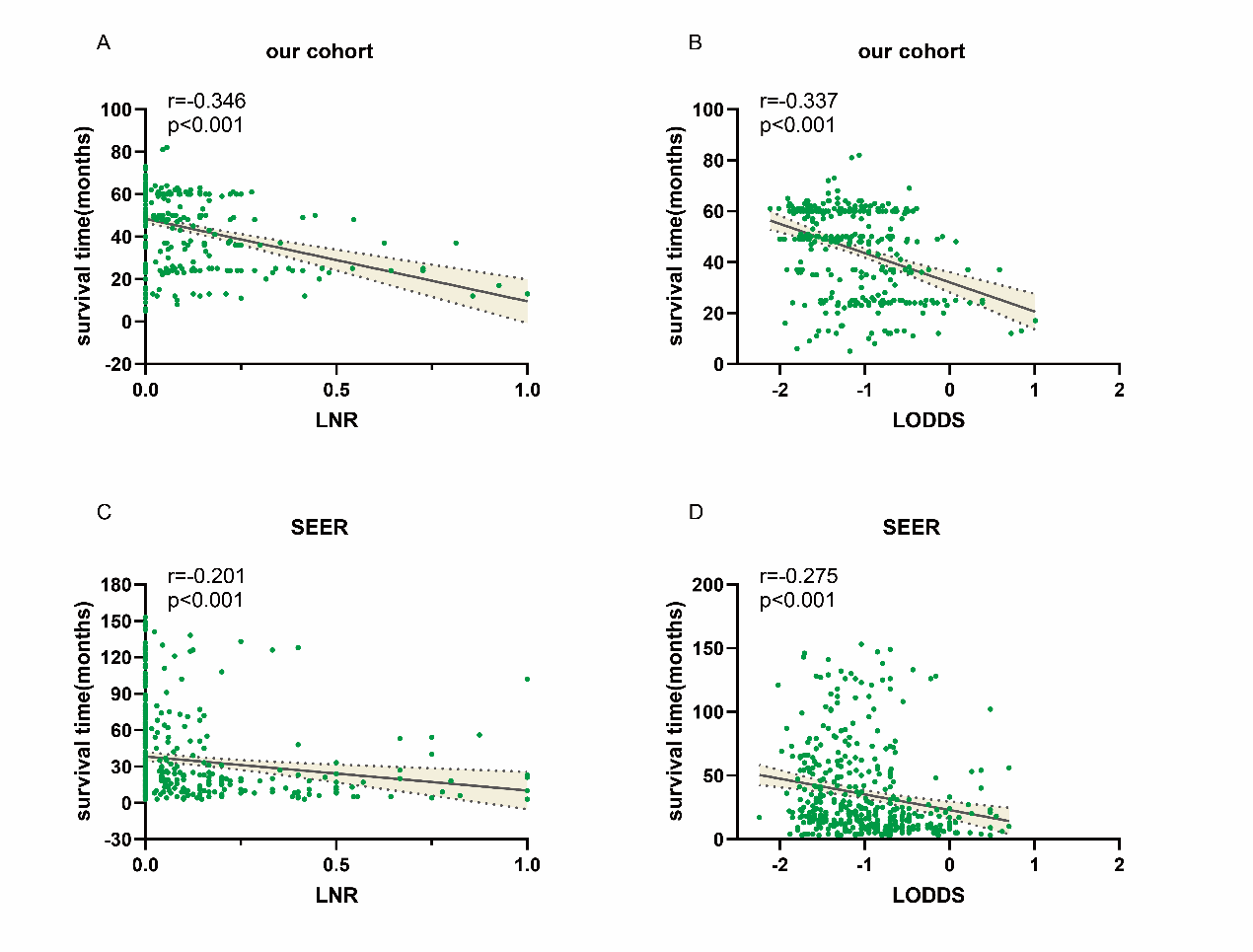
**

**Supplementary Figure 1** Correlation analysis: Survival time and LNR (A, C); Survival time and LODDS (B, D).


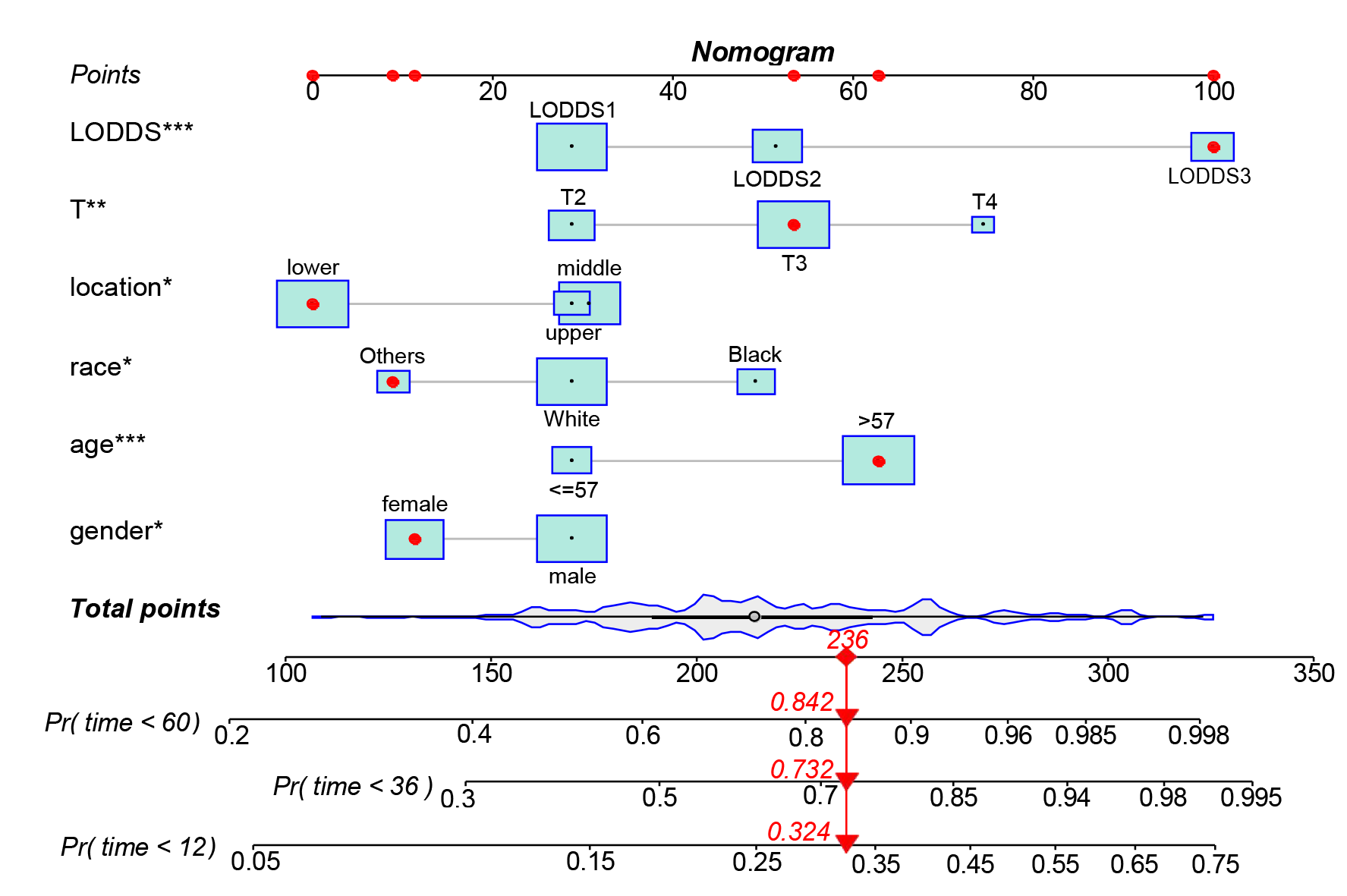


**Supplementary Figure 2** A nomogram for predicting the 1-, 3-, and 5-year OS for advanced ESCC patients from SEER dataset.

**Supplementary Table 1** Multivariate Cox regression analysis for OS and predictive performance of different LNs models in the validation set(N=409).

| Variable | ModelⅠ(N) | | ModelⅡ(LNR) | | Model Ⅲ (LODDS) | | Model Ⅳ | |
| --- | --- | --- | --- | --- | --- | --- | --- | --- |
|  | HR(95%CI) | P-value | HR(95%CI) | P-value | HR(95%CI) | P-value | HR(95%CI) | P-value |
| Gender |  | 0.092 |  | 0.081 |  | 0.040 |  | 0.062 |
| Male | Ref |  | Ref |  | Ref |  | Ref |  |
| Female | 0.819 (0.649-1.033) |  | 0.812 (0.642-1.026) |  | 0.784 (0.622-0.989) |  | 0.799 (0.631-1.011) |  |
| Age (years) |  | 0.001 |  | 0.001 |  | 0.001 |  | 0.001 |
| ≤57 | Ref |  | Ref |  | Ref |  | Ref |  |
| >57 | 1.642 (1.237-2.180) |  | 1.649 (1.244-2.185) |  | 1.600 (1.207-2.120) |  | 1.589 (1.194-2.114) |  |
| Race |  | 0.004 |  | 0.005 |  | 0.026 |  | 0.034 |
| White | Ref |  | Ref |  | Ref |  | Ref |  |
| Black | 1.444 (1.088-1.915) |  | 1.422 (1.069-1.891) |  | 1.328 (0.996-1.770) |  | 1.299 (0.967-1.744) |  |
| Others | 0.748 (0.521-1.073) |  | 0.737 (0.514-1.056) |  | 0.759 (0.530-1.089) |  | 0.745 (0.517-1.074) |  |
| Tumor location |  | 0.005 |  | 0.002 |  | 0.001 |  | 0.001 |
| Upper | Ref |  | Ref |  | Ref |  | Ref |  |
| Middle | 0.992 (0.693-1.421) |  | 1.038 (0.727-1.482) |  | 1.027 (0.719-1.465) |  | 1.028 (0.718-1.470) |  |
| Lower | 0.682 (0.481-0.967) |  | 0.680 (0.480-0.961) |  | 0.671 (0.474-0.950) |  | 0.662 (0.467-0.938) |  |
| T stage |  | 0.008 |  | 0.012 |  | 0.014 |  | 0.015 |
| T2 | Ref |  | Ref |  | Ref |  | Ref |  |
| T3 | 1.482 (1.118-1.965) |  | 1.429 (1.077-1.897) |  | 1.409 (1.062-1.871) |  | 1.417 (1.066-1.885) |  |
| T4 | 1.858 (1.157-2.983) |  | 1.861 (1.161-2.982) |  | 1.879 (1.171-3.016) |  | 1.844 (1.149-2.961) |  |
| N stage |  | 0.001 |  |  |  |  |  | 0.949 |
| N0 | Ref |  |  |  |  |  | Ref |  |
| N1 | 1.318 (1.026-1.694) |  |  |  |  |  | 0.844 (0.437-1.631) |  |
| N2 | 1.851 (1.285-2.667) |  |  |  |  |  | 0.907 (0.480-1.713) |  |
| N3 | 2.397 (1.283-4.481) |  |  |  |  |  | 0.983 (0.439-2.201) |  |
| LNR |  |  |  | <0.001 |  |  |  | 0.382 |
| LNR0 |  |  | Ref |  |  |  | Ref |  |
| LNR1 |  |  | 0.773 (0.460-1.299) |  |  |  | 0.958 (0.419-2.189) |  |
| LNR2 |  |  | 1.381 (1.049-1.817) |  |  |  | 1.359 (0.728-2.540) |  |
| LNR3 |  |  | 2.339 (1.703-3.214) |  |  |  |  |  |
| LODDS |  |  |  |  |  | <0.001 |  | 0.001 |
| LODDS1 |  |  |  |  | Ref |  | Ref |  |
| LODDS2 |  |  |  |  | 1.374 (1.048-1.801) |  | 1.220 (0.858-1.737) |  |
| LODDS3 |  |  |  |  | 2.657 (1.976-3.573) |  | 2.820 (1.611-4.936) |  |
| C-index | 0.635 |  | 0.653 |  | 0.655 |  |  |  |
| LRχ2 | 60.59 |  | 74.06 |  | 83.76 |  |  |  |
| -2LLR | 3269.58 |  | 3256.73 |  | 3247.71 |  |  |  |
| AIC | 3281.03 |  | 3267.57 |  | 3255.87 |  |  |  |
| CI: confidence interval; HR: hazard ratio; C-index: Harrell’s concordance index; LRχ2: the likelihood ratio χ 2 score; -2LLR: the (-2) log-likelihood ratio;  AIC: the Akaike information criterion. | | | | | | | | |

**Supplementary Table 2 Subgroup analysis stratified by the LODDS system in the validation set.**

| Variable | Total | LODDS1 | LODDS2 | LODDS3 | Chi-square | P value |
| --- | --- | --- | --- | --- | --- | --- |
| Total | 409 | 223 (54.5) | 107 (26.2) | 79 (19.3) |  |  |
| Gender |  |  |  |  | 1.563 | 0.458 |
| Male | 243 (59.4) | 128 (57.4) | 69 (64.5) | 46 (58.2) |  |  |
| Female | 166 (40.6) | 95 (42.6) | 38 (35.5) | 33 (41.8) |  |  |
| Age(years) |  |  |  |  | 0.779 | 0.678 |
| ≤57 | 99 (24.2) | 53 (23.8) | 24 (22.4) | 22 (27.8) |  |  |
| >57 | 310 (75.8) | 170 (76.2) | 83 (77.6) | 51 (72.2) |  |  |
| Race |  |  |  |  | 11.476 | 0.022 |
| White | 276 (67.5) | 157 (70.4) | 69 (64.5) | 50 (63.3) |  |  |
| Black | 74 (18.1) | 28 (12.6) | 27 (25.2) | 19 (24.1) |  |  |
| Others | 59 (14.4) | 38 (17.0) | 11 (10.3) | 10 (12.7) |  |  |
| Tumor location |  |  |  |  | 2.264 | 0.687 |
| Upper | 52 (12.7) | 30 (13.5) | 14 (13.1) | 8 (10.1) |  |  |
| Middle | 154 (37.7) | 89 (39.9) | 38 (35.5) | 27 (34.2) |  |  |
| Lower | 203 (49.6) | 104 (46.6) | 55 (51.4) | 44 (55.7) |  |  |
| T stage |  |  |  |  | 0.213* | 0.012 |
| T2 | 110 (26.9) | 72 (32.3) | 26 (24.3) | 12 (15.2) |  |  |
| T3 | 270 (66.0) | 134 (60.1) | 75 (70.1) | 61 (77.2) |  |  |
| T4 | 29 (7.1) | 17 (7.6) | 6 (5.6) | 6 (7.6) |  |  |
| N stage |  |  |  |  | 0.818* | <0.001 |
| N0 | 226 (55.3) | 180 (80.7) | 37 (34.6) | 9 (11.4) |  |  |
| N1 | 126 (30.8) | 43 (19.3) | 51 (47.7) | 32 (40.5) |  |  |
| N2 | 45 (11.0) | 0 (0.0) | 18 (16.8) | 27 (34.2) |  |  |
| N3 | 12 (2.9) | 0 (0.0) | 1 (0.9) | 11 (13.9) |  |  |
| LNR |  |  |  |  | 0.868* | <0.001 |
| LNR0 | 226 (55.3) | 180 (80.7) | 37 (34.6) | 9 (11.4) |  |  |
| LNR1 | 26 (6.4) | 26 (11.7) | 0 (0.0) | 0 (0.0) |  |  |
| LNR2 | 95 (23.2) | 17 (7.6) | 70 (65.4) | 8 (10.1) |  |  |
| LNR3 | 62 (15.2) | 0 (0.0) | 0 (0.0) | 62 (78.5) |  |  |
| *:Gamma value, two-way ordered chi-square test | | | | | | |
